# Women’s experiences of ovulation testing: a qualitative analysis

**DOI:** 10.1186/s12978-015-0103-y

**Published:** 2015-12-21

**Authors:** Georgina Jones, Jill Carlton, Sarah Weddell, Sarah Johnson, William L. Ledger

**Affiliations:** Health Economics and Decision Science (HEDS), School of Health and Related Research (ScHARR), University of Sheffield, Regent Court, 30 Regent Street, Sheffield, S1 4DA UK; Scientific and Medical Affairs, SPD Development Company Limited, Clearblue Innovation Centre, Priory Business Park, Bedford, MK44 3UP, UK; Obstetrics and Gynaecology, University of New South Wales, Sydney, NSW 2052 Australia

**Keywords:** Ovulation testing, Infertility, Qualitative methods, Conception, Telephone interviews

## Abstract

**Background:**

The introduction of home digital ovulation tests (OTs) has provided a simple solution for women wishing to optimise the timing of intercourse when trying to conceive. However, despite this, very little is understood about women’s experiences of using these tests.

**Methods:**

We carried out qualitative, semi-structured telephone interviews with women who were seeking to conceive (not actively undergoing clinical investigation/fertility treatment) from the general UK population. The interviews were conducted following participation in a randomised controlled trial (RCT) in which participants were either provided with digital home OTs to assist in timing intercourse (*n* = 18) or advised to have intercourse every 2–3 days (*n* = 18). The interviews were digitally recorded, transcribed and then analysed using Framework analysis to identify the themes.

**Results:**

Data saturation was reached after 36 interviews. The use of the OT appeared to elicit 10 key themes, which could be described within the context of three overarching issues: 1) a positive impact (understanding the menstrual cycle, confirming when ovulating, emotional support, improving the relationship), 2) a negative impact (changing sex life and relationship with their partner, the emotional consequences of prolonged use, questions and uncertainty about what their results mean for them) and 3) the experiences of trying to conceive in general (use of clinical guidance and emotional experience).

**Conclusions:**

Overall, the use of home OTs were found to affect women’s thoughts and feelings in multiple ways during attempts to conceive. Although some women reported a range of negative experiences when using OTs, they also reported similar negative experiences when trying to conceive without using the tests. However, there were many positive themes associated with OT use, including an increased understanding of the menstrual cycle, confirmation of ovulation timing and providing a source of help and support when trying to conceive. Overall, when women are trying to conceive, ensuring they have access to high-quality information, including use of OT, may be of benefit to help address some of the questions and uncertainties that were raised by the participants in this study.

**Trial registration number:**

NCT01084304

**Electronic supplementary material:**

The online version of this article (doi:10.1186/s12978-015-0103-y) contains supplementary material, which is available to authorized users.

## Background

It is estimated that one in six couples in the UK are suffering from infertility and difficulties in conceiving. Infertility and reproductive failure has been identified as a major life stressor [1], and numerous studies have reported on the psychological and emotional consequences this can have upon the lives of the women and couples affected by this condition [2]. Failure to become pregnant is likely to be the greatest cause of stress when a woman is trying to conceive [3].

Research has shown that conception is most likely when intercourse takes place in the 5 or 6 days leading up to ovulation [4] and that the clinical prediction that ovulation usually occurs within days 10–17 of the menstrual cycle is often incorrect [4–7]. Recent evidence has also shown that many women have a poor understanding of their menstrual cycle and the process of ovulation [8]. Incorrect timing of intercourse is therefore thought to be a common but easily correctable cause of difficulty in conceiving.

A variety of techniques are used by women to predict ovulation, some more accurate than others [9], including calendar methods, basal body temperature measurement, examination of cervical mucus and home ovulation tests (OTs), which detect the surge in luteinising hormone (LH) that precedes ovulation. The advancement in the ease of use and accuracy of home OTs, in particular the introduction of digital tests, has provided a simple solution for women wishing to optimise the timing of intercourse. Despite this, it has been suggested that timing intercourse to coincide with ovulation by using OTs, or other fertility awareness-based methods, can lead to emotional distress. A previous retrospective observational study by Kopitzke et al. of 26 patients undergoing infertility treatment has often been cited as providing evidence that OTs induce stress, as patients in this study reported an increase in stress when using the tests. However, these patients reported that events associated with lack of conception or loss of pregnancy, such as a negative pregnancy test, onset of menses, ectopic pregnancy or miscarriage and even seeing a pregnant woman, were in fact emotionally more difficult than using the OTs [10].

A recent randomised controlled trial (RCT) by Tiplady et al. directly examined whether OT usage in women seeking to conceive elicited stress (NCT01084304) [11]. Two hundred and ten women seeking to conceive, but not under clinical supervision or fertility investigation, were recruited from the general UK population. All women were living in the UK, aged between 18 and 40 years, having regular menstrual bleeds and wishing to become pregnant. Women were excluded if they were currently undergoing fertility treatment or investigation, had previously been diagnosed as infertile, had a history of depression, anxiety or panic attacks or were dependent on drugs or alcohol. Women who had previously used ovulation tests were not excluded from participating in the study. One hundred and fifteen participants were randomised to the test group (provided with digital home OTs to time intercourse to coincide with their most fertile period) and 95 to the control group (advised to have intercourse every 2–3 days) through block randomisation. Questionnaire- and biomarker-based data were collected from both groups, and women participated in the study for two complete consecutive menstrual cycles.

As part of this RCT, qualitative individual telephone interviews, using both fixed and open-ended questions, were also carried out with a purposive sample of 101 participants to gain feedback on product use and more detail on their experiences of using the OT. A random sample from only 30 participants was analysed for the fixed-theme questions and reported in the aforementioned paper by Tiplady et al. [11]. The main benefit of using OTs was reported as their ability to pinpoint when ovulation occurs, helping participants focus their conception attempts. This provided some – albeit incomplete – insight into the qualitative elements of emotional well-being when using OTs.

The aim of this study was to undertake a more in-depth analysis of these qualitative interviews and, in particular, to apply a rigorous thematic assessment of the open-ended questions put to study participants. It was anticipated that this would provide a deeper understanding of the experiences of using OTs of women who were not under investigation for infertility but were wishing to conceive.

## Methods

The individuals interviewed for this study were participants in a prospective RCT to assess whether the use of a digital home OT had any effect on the level of stress in women seeking to conceive [11]. In total, 210 women were recruited via an advert placed on the Clearblue UK website. All participants were aged 18–40 years, were experiencing regular menstrual bleeds and were wishing to conceive. They were randomised to use a digital OT to time intercourse to coincide with the most fertile period of their menstrual cycle or provided with the UK National Institute for Health and Clinical Excellence (NICE) guidelines for increasing the chance of conception (intercourse every 2–3 days). The study lasted for the duration of two complete consecutive menstrual cycles for each participant. However, participants in the control arm were also sent the digital OT to use at the end of their two complete menstrual cycles, and therefore at the time of interviewing, most participants had experience of using the digital OT regardless of initial randomisation. The digital OT used in this study (Clearblue Digital Ovulation Test, SPD Development Company, Bedford, UK) tracks the changing levels in urinary LH. When the device detects a surge in LH, it displays a ‘smiley face’ to indicate that the user is at her peak level of fertility for that cycle. Ethics approval was obtained from the Institutional Review Board where this study took place (trial registration number: NCT01084304).

**Fig. 1:**
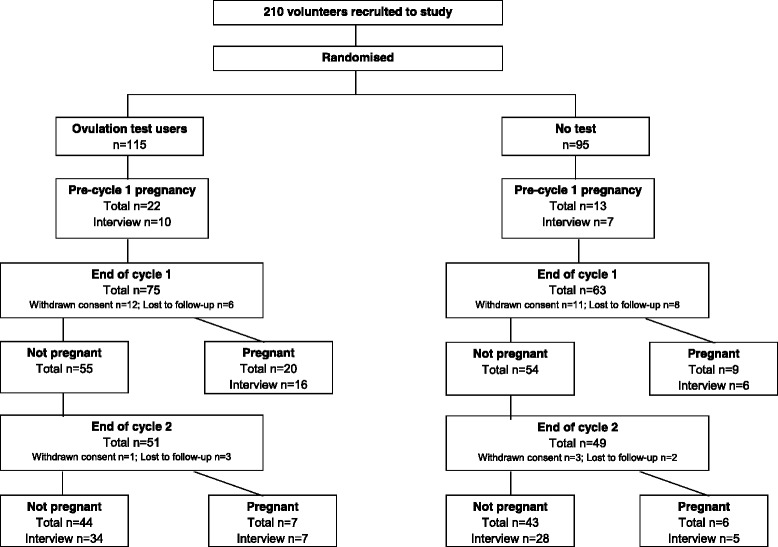
Flow diagram indicating the number of participants eligible for interview and the number of interviews that were conducted at each point in the study. N.B. Participants who could not be contacted within 2 months of the end of the study were not interviewed

Interviews with study participants were conducted following two complete menstrual cycles after randomisation (study end) or after a pregnancy had been reported. All participants were asked to give their opinions based on their experience of using OTs at the point of interview. All participants were invited for interview, from both arms of the study and with different pregnancy outcomes, to ensure that the full range of possible emotions experienced when using the OT were identified. All participants contacted after the study agreed to be interviewed; however, those who could not be contacted within a reasonable time period (2 months) from the end of the study were not interviewed. The numbers of participants eligible for interview along with the number of interviews that were conducted in each study group and for each study outcome are shown in Fig. 1. The interviews were carried out by telephone, by a single interviewer using a semi-structured schedule that comprised both closed (fixed responses) and open questions. The interviewer had received formal training on how to conduct structured interviews.

Telephone interviews were transcribed verbatim and the transcripts imported into QSR NVivo 9^©^ (QSR International, Doncaster, Australia). The open-ended questions used in the semi-structured interview schedule are reported in Table 1. The analysis of these was guided by the research question “How does the use of home ovulation tests affect women?” and the interviews were analysed as a whole, not by initial group of randomisation, as the overarching aim of the study was to explore participants’ experiences of using the digital OT overall and not to compare the emergent themes by randomisation group. Thematic content analysis was undertaken using Framework (an approach developed by the National Centre for Social Research) which follows the principles of classifying and organising data according to key themes, concepts and emergent categories [12]. To initiate analysis, a small sample of interviews from the start, middle and end of the study were selected to overcome the possibility that familiarisation with the interviews and data may have influenced the nature and style of the interview (*n* = 8).

**Table 1:** Summary of the semi-structured open-ended Interview Schedule

Question
Tell us about your experience in trying to conceive prior to joining the study?
How do you now feel about trying to conceive having completed the study?
Prior to joining this study, did your cycles vary in length, and do you think you knew when you were ovulating? If so, how did you know?
What did/do you expect the Clearblue Digital Ovulation Test to give you?
Did the ovulation test help you to notice any changes in your body during your cycle?
How did you feel when you did/did not see the smiley face result?
Did you feel any pressure or stress when using the ovulation test?
Did you follow the NICE guidelines for achieving conception? If so explore.
Do you feel any pressure or stress during your cycle? If so explore when?
Did using the ovulation test change your behaviour in any way?
Did using the ovulation test change the intimacy between yourself and your partner? If so, explore how?
What do you think have been/will be the main advantages of ovulation test use?
What do you think have been/will be the main disadvantages of ovulation test use?

Each transcript was reviewed several times by a single researcher (JC), in order for them to become familiar with the data. Key phrases and sentences were identified that related to the experiences of participants when trying to conceive. Emergent themes were identified and an initial coding framework developed. A second researcher (GJ) reviewed 50 % of these transcripts (*n* = 4). There was absolute agreement between the researchers regarding the initial coding framework, but it was felt to be too brief. Therefore the resulting primary coding framework was more in-depth and included more sub-themes to support the analysis of the data. Following this, the initial eight transcripts along with a further 28 transcripts (also taken from the start, middle and end of the study) were then re-examined and coded according to the more detailed themes identified by JC. In total, 30 % of the 36 full transcripts were checked for coding consistency by the second researcher (GJ).

## Results

Data saturation was reached after the analysis of 36 transcripts using the more in-depth primary coding framework, with 18 participants from the control group and 18 from the test group included. The groups were balanced, with approximately the same number of participants from each group achieving pregnancy within the study period (Table 2). Individual volunteer demographics and unique identification numbers are provided as (Additional file 1: Table S1). The mean age of the participants was 30 years (range: 22–39 years); a summary of other volunteer demographics is provided in Table 2.

**Table 2:** Summary of volunteer demographics (individual volunteer characteristics are reported in Additional file 1: Table S1)

	Control group	Test group
Number of volunteers	18	18
Age range, years	22–39	25–38
Number of previous pregnancies, *n* (%)		
0	1 (5.6)	5 (27.8)
1	9 (50)	6 (33.3)
2	7 (38.9)	5 (27.8)
3	1 (5.6)	1 (5.6)
6	0 (0.0)	1 (5.6)
Number of live births, *n* ^a^ (%)		
0	5 (27.8)	9 (50)
1	9 (50)	5 (27.8)
2	3 (16.7)	4 (22.2)
Number of miscarriages, n^b^ (%)		
0	9 (50)	13 (72.2)
1	8 (44.4)	2 (11.1)
2	0 (0.0)	1 (5.6)
5	0 (0.0)	1 (5.6)
Number of months trying to conceive prior to study entry, *n* (%)		
≤ 3 months	5 (27.8)	6 (33.3)
4–6 months	6 (33.3)	7 (38.9)
7–12 months	6 (33.3)	2 (11.1)
≥ 13 months	1 (5.6)	3 (16.7)
Previous use of ovulation kits, *n* (%)		
Yes	11 (61.1)	9 (50.0)
No	7 (38.9)	9 (50.0)
Pregnancy within the study period^c^ (%)		
Yes	9 (50.0)	7 (38.9)
No	9 (50.0)	11 (61.1)

^a^Information not available for 1 control group volunteer

^b^Information not available for 1 control and 1 test group volunteer

^c^PTP: pre-trial pregnancy for 2 control and 1 test group volunteer (women became pregnant after consenting to participate in the study, but prior to participating in the study)

Ten key themes were identified from the analyses, which fell into three overarching themes. These were the positive (Table 3) and negative (Table 4) impacts of using OTs, both on participants and their partners and the participants’ narratives concerning attempting to become pregnant in general (Table 5). As mentioned previously, most participants had used the digital OT by the time of interviewing, regardless of initial randomisation. As the participants described similar experiences of using the digital OT, the 10 themes have been presented to aid the reader’s understanding of participants’ experiences overall and therefore without reference to the initial group of randomisation. The results have been reported under each of the 10 main themes; the sub-themes are in italics in the text, and supporting sample quotes are also reported in Tables 3, 4 and 5.

**Table 3:** Positive themes, sub-themes and example quotes from telephone interviews about use of ovulation tests

Themes	Sub-themes	Sample quote (participant ID number)^a^
1. Understanding the menstrual cycle	1.1 Increased knowledge	“*Looking on it now in hindsight*, *no I didn*’*t know my cycle at all*.” (3)
	1.2 Digital charting/tracking	“*I wanted to just really get to know what my cycle was and see if that would be helped by the ovulation tests. You sometimes also have those sorts of concerns like is there something more that you need to be doing as opposed to trying to chart it manually*.” (10)
	1.3 Precision	“*I was interested to see how charting*, *especially when I got the information*, *how my emotions would affect everything and to actually measure my cycle a lot more precisely*.” (1)
	1.4 Surprise	“*I think what the study has certainly highlighted to me is the differences across my own cycles and the ovulation days are quite different on a month to month basis*.” (10)
2. Confirmed when Ovulating	2.1 Accuracy of ovulation	“*I sort of knew it happened within a week from 10*–*14 days but obviously my cycles were a little bit different ranging from like 25 to 28 days so it varied every day*, *every month sort of thing*…*sometimes I ovulated earlier than I thought and then other times I ovulated later so it was good to get an accurate day*.” (9)
	2.2 Pinpointing ovulation	“*They pinpoint the right time to try*, *and it kind of takes that pressure off*; *it takes the guesswork out of it really*; *you don*’*t have to look for other signs as well*.” (24)
	2.3 Planning conception	“*I don*’*t think it got easier because every month you had an expectation and when obviously you hadn*’*t conceived it had an impact*, *but it was almost balanced out because you were disappointed that you hadn*’*t conceived*, *but at the same time you were able to say*, ‘*Right*, *next month*’, *and you had a timeline and you were able to plan it more effectively*.” (10)
	2.4 More relaxed	“*Just knowing*, *I think it*’*s just nice to know that you are timing things right. I think it*’*s just nice to know that you are at the right day and things*, *and the right time*, *it makes you feel a bit more relaxed about everything I think*.” (8)
	2.5 Noticing physical changes	“*It just makes you more aware of your fertile times*, *of your cervical mucus and things like that. I think it just makes you just generally more aware of what*’*s going on with your body and things*.” (23)
3. Emotional support	3.1 Reassurance	“*It made me much more comfortable that everything was still ok* … *and it also reassured me that a woman in her late 30s still can conceive*.” (10)
	3.2 Helping hand (to get pregnant)	“*Well*, *just sort of the timing and things really. I think if you time it better then it*’*s more likely to increase your chances of conceiving*.” (23)
	3.3 Helping hand (alert something wrong)	“*But I suppose that*’*s a positive thing because you could then go to a doctor*, *some women go on for years without realising*.” (17)
	3.4 Smiley face	“*Yes*, *he was quite excited about the face as well*. ‘*Oh*, *show me the face*!’ *And it was great*, *on the test*, *and he*’*d go all shy*, ‘*Oh*, *I can*’*t believe that face*!’” (22)
4. Improving the relationship	4.1 Less stressful	“*The main reason is that you know your exact time of your ovulation. And it just keeps you stress free from trying to guess*, ‘*Oh is it the sign or is it the time*?’ *You know*, *it makes the whole process much less stressful and easier for couples*.” (22)
	4.2 Teamwork	“*We have used them this month. We have enjoyed using them this month and it*’*s kind of like a joint effort. I get asked if I have used my sticks yet and is it the right time*, *to me it*’*s like a project*.” (1)

^a^Participant demographic details are listed against their ID number in Additional file 1: Table S1

**Table 4:** Negative themes, sub-themes and example quotes from telephone interviews about use of ovulation tests

Themes	Sub-themes	Sample quote (participant ID number)^a^
5. Sexual life	5.1 Manufactured	“*Well it*’*s [intercourse] more intense between the middle of my cycle than actually all over the place like it used to be… It somehow became a bit technical.”* (6)
	5.2 No excitement	*“A bit mechanical I guess rather than having fun and just being relaxed. I suppose it just took the fun out of it.”* (18)
	5.3 I missed it!	*“I did another test when he came from work and I didn’t have this face and I was so upset. I started ringing the helpline and I said, ‘I’m afraid I missed it; he was at work and I couldn’t do anything’.”* (22)
	5.4 Raised expectations	*“I just thought I’d fall quicker.”* (18) *“I was very excited to see it, and then automatically thought I might fall pregnant that month, so it was sort of a high when you saw the face and a low at the end of the month.”* (25)
6. Relationship with partner	6.1 Pressure	*“It can increase the pressure between the couple. Obviously, if you both know that day is the day, sort of thing, it sort of increases the pressure a little bit. Although I didn’t find that but my husband said he did*.” (23)
	6.2 Less romantic	*“…it killed the romance a bit.”* (6)
	6.3 Secretive	*“I sort of thought not to tell him.”* (23)
7. Emotional consequences of prolonged use	7.1 Dependence	“Y*ou could get too emotionally dependent on them if you are really desperate and they are the only thing that you could afford to do at the time.”* (2)
	7.2 Stressed	*“Well I didn’t fall pregnant on it. My boyfriend hated them, he thought it made me a stress head.”* (18)
	7.3 Obsession	*“You’re waiting for that happy, smiley face to come, I found that I was getting a bit obsessed by it all, to be honest.”* (25)
	7.4 Artificial	*“He didn’t mind but as I say, you know, he was quite happy for me to do it but then the more and more times I kept on doing it he was like, you know, stop relying on it, you should do it naturally.”* (9)
8. Questioning and uncertainty	8.1 Self doubt	*“Occasionally you get the odd stick which didn’t work but it might have been just the way you’re holding it or whatever.”* (11)
	8.2 Blame	*“I thought what have I done, I have done something wrong……..at once, I thought it was a user problem as opposed to the test problem.”* (10)
	8.3 Confused why not working?	*“Only when I say my period arrived, that’s the only time I was a bit stressed. I was like, you know, why isn’t it working, the test is saying this is when, we’re doing it when it’s telling us to and obviously there was no result after it.”* (9)
	8.4 Demotivating	*“If the test says you are ovulating but you don’t conceive then I suppose it can be a bit de-motivating I suppose. Just a bit more frustrating.”* (2)
	8.5 Failure	“*I suppose it’s like when you get your period that’s when I feel more stressed about anything because you kind of see it as another month that you’ve failed.*” (36)
	8.6 Something more wrong	*“It was really easy and straightforward to use, the only thing was that from day six, there was similar disappointment to a pregnancy test to not getting a smiley face, and that was quite negative, so I was thinking oh god is it me and then my mind was going do I not ovulate? Is that why I haven’t fallen pregnant for all this time?”* (17)

^a^Participant demographic details are listed against their ID number in Additional file 1: Table S1

**Table 5:** Trying to conceive in general themes, sub-themes and example quotes from telephone interviews about use of ovulation tests

Themes	Sub-themes	Sample quote (participant ID number)^a^
9. NICE guidance	9.1 Unrealistic expectations	*“I know the NHS say you are supposed to try every other day, but sometimes, that’s just not possible, for lots of different reasons”.* (2)
	9.2 No energy	*“It kind of makes sense, sometimes it’s a bit more difficult, when you are both really, really tired, if you do work you know.”* (1)
	9.3 No inclination	*“Having intercourse every other day is just not possible, we don’t have the inclination either.”* (2)
	9.4 Pressure on relationship	*“Yes we did have sex every other day, it put quite a lot of pressure on your relationship, because you are not doing intercourse for enjoyment, only to try for a baby.”* (7)
	9.5 Sex as a chore	*“Yes, every other day, it becomes a bit of a chore rather than a pleasure after a while.”* (26)
	9.6 Not enjoyable	*“I was a bit fed up at the end, we both said that, last month we decided we weren’t going to be like that, it was too clinical, it wasn’t fun, because at the end of the day although you want an outcome, if it isn’t fun there is no point and at the end it was like that too clinical.”* (28)
	9.7 Guessing	*“Just taking a stab in the dark.”* (7)
10. Emotional experience	10.1 Why is it not happening?	*“It wasn’t because of the study, and I think I would have felt like that anyway. I mean we are still trying for a baby and every time I get my period I feel frustrated and annoyed, so, no I don’t think it was really any extra pressure from what I feel anyway.”* (17)
		*“It [the OT] takes out the spontaneity and puts pressure on your partner, but then that’s true of any type of fertility tracking, whether its temperature or mucus or whatever.”* (19)
	10.2 What will be will be	*“Because life sometimes gets in the way, we both work full time and you know you think if it’s going to happen, it’s going to happen.”* (2)

^a^Participant demographic details are listed against their ID number in Additional file 1: Table S1

### Positive themes (Table 3)

1. Understanding the menstrual cycle (sub-themes in italics)
  One of the main positive themes reported by most participants who had used the OT was that they had increased *knowledge* of their menstrual cycles compared with before using the OT. Prior to using the tests, there was the perception that the digital nature of the OT would enable them to *chart and track* their own cycle in a different way to other methods (e.g. manual charts). Many of the participants’ experiences supported this, reporting that the OT had enabled them to connect with more *precision* the emotions they experienced at different times during their menstrual cycle. For some participants, this new understanding of the menstrual cycle had also caused *surprise* regarding how much variation there was within their own cycles, which they had not been aware of before using the OT.

2. Confirming when ovulating (sub-themes in italics)
  The main way in which participants had gained more knowledge about their menstrual cycle was that the OT had confirmed to them when they were ovulating. This was described as the main advantage of the OT by almost all participants in this study. They described particularly liking the *accuracy* with which ovulation was detected. Some participants had initially used the OT to increase their general awareness of their menstrual cycle and fertile period. However, other participants reported a desire to be able to *pinpoint* their most fertile days with precision, which they had successfully done, and as a result had found that the OT had enabled them to *plan conception* more effectively. Participants reported that being able to identify their fertile period made them feel less stressed and improved their emotional well-being. This was reported from a personal perspective, where participants felt that they were more *relaxed* as they knew they were ovulating (and there was no problem “with them”). Some participants reported that they had been aware of when they ovulated because of changes in their body (such as increased discharge or breast tenderness), but that the OT confirmed this and made them *notice the physical changes* around their time of ovulation more.

3. Emotional support (sub-themes in italics)
  Participants described an emotional connection to the test. In one sense, this was because it helped alleviate concerns and negative emotions associated with failing to conceive. Participants described that they felt *reassurance* and found it “comforting” to know that they were at least ovulating. Participants appeared to have the belief that the OT was a helpful aid. This was observed in two ways: firstly, they believed it provided a ‘*helping hand*’ *towards becoming pregnant*; secondly, for some participants who failed to detect ovulation when using the OT, they also felt that the test had provided a *helping hand to alert them sooner that something might be wrong*, thus enabling them to seek additional advice and support. However, this emotional connection also appeared to be a consequence of the ‘*smiley face*’ which is displayed to tell the user when she is ovulating. One participant described feeling “happy and giddy to see a smiley face” and this emotional connection was not exclusive to the participants but applied to their partners too.

4. Improving the relationship (sub-themes in italics)
  Using the OT did have an impact upon the relationship between some participants and their respective partners, in both a positive and negative manner. One of the positive areas that participants reported was because the OTs allowed accurate monitoring of their fertile period, they said they felt *less stress* and pressure in trying to conceive. They (and their partner) could identify that they were on the right track for trying, and they could have intercourse during the fertile period. Some participants reported that using the OTs made their partner more interested in the conception process; the OTs gave them the feeling that they were working *together as a team*.

### Negative themes (Table 4)

5. Impact upon sex life (sub-themes in italics)
  On the other hand, knowing when their fertile period was meant that some participants felt compelled to have intercourse at certain times, and that their sex life became more *manufactured* and less fun, with *no excitement*. The OT also created some anxiety about not being able to have intercourse at the right time, with a few participants expressing feeling upset that they might have *missed* their opportunity to conceive if they were ovulating but their partner wasn’t around. This was compounded by the fact that many of the participants thought they would become pregnant more quickly when using the digital OT than if they hadn’t used it, and thus using the OT *raised their expectations* of conceiving.

6. Intimate relationship with their partner (sub-themes in italics)
  Whereas some participants appeared to feel anxious about not having intercourse at the optimal time, partners were described as feeling more *pressure* to perform sexually. The participants believed that this could/did increase the pressure on their partner and, therefore, the relationship was typically described as becoming *less romantic*. To avoid this, a small number of participants reported that they chose not to disclose when they had identified their fertile period and kept this knowledge as *a secret* from their partner.

7. Emotional consequences of prolonged use (sub-themes in italics)
  A concern of some participants (and their partners) was the potential feeling of *dependence* upon the OT itself that they anticipated may occur with prolonged use. This led to a small number of participants describing putting ‘pressure’ on themselves and finding it was “*stressful*” because using the OTs could become an *obsession* if used over a long period of time. Participants also described their partners not liking the *artificial* nature of using the test to conceive.

8. Questions and uncertainties (sub-themes in italics)
  For some participants, there was a degree of *self*-*doubt* associated with using the OT, and whether a negative result was related to not ovulating, or whether they had performed the test correctly. However, for other participants this went deeper, with some *blaming* themselves if the test didn’t work. This seemed to be a particular issue for those who were ovulating and yet failing to conceive, as they had expected to conceive when the OT result was positive and they had timed intercourse within their fertile period. As a result of this they found it *confusing* not to conceive, which seemed most evident when the participants’ period arrived, thus proving this to be a stressful time in the cycle. Whereas some participants were able to maintain feeling positive and plan for the next month, for others the impact was more severe, as they described feeling *demotivated* and also like a *failure*. There were also questions and uncertainties from participants who had a negative test result, for example worrying whether *something more might be wrong*.

### Trying to conceive generally (Table 5)

9. NICE Guidance (sub-themes in italics)
  Most of the participants in the study were aware of the NICE guidance recommending intercourse every 2–3 days. However, those who had used this method to conceive felt that it was *unrealistic* for most couples. Many participants described having not enough *energy* to meet this guidance or that they had *no inclination* to, mainly as a result of feeling too tired. They also felt that the advice on aiming to have intercourse every other day placed an increased *pressure on the relationship*. In this instance, participants described intercourse as *less enjoyable*, a *chore*, and mechanical under these circumstances. This was also compounded by the feeling that they were *guessing* when the best time to conceive might be.

10. Emotional experience (sub-themes in italics)
  When participants were asked about their thoughts and experiences of trying to conceive, most reported that it was an emotional and stressful time, and acknowledged that this could have been experienced whether using the OTs or not. One participant described feeling surprised that she didn’t become pregnant naturally first time, because this was how women are told it will happen when they are younger. Most participants described the phase leading up to their period as a stressful time, waiting to see whether it had worked/happened, and described the arrival of their period as a time of *frustration when nothing had happened*, although some expressed a more fatalistic viewpoint towards conceiving (i.e. “*What will be*, *will be*”).

All themes were discussed by participants regardless of whether or not they achieved a pregnancy during the study period. Sub-themes associated with the effect of using OT on their sex life were discussed far less by participants who achieved a pregnancy compared with those who did not. Discussion of the positive themes, particularly those relating to understanding of the menstrual cycle and confirmation of ovulation, were very common among participants and were based on their actual experiences. In contrast, some of the negative themes such as emotional consequences of prolonged use were far less commonly discussed and were often based on perception rather than on actual experience.

## Discussion

The aim of this study was to use qualitative methods to explore in-depth women’s experiences of using an ‘over-the-counter’ digital home OT when trying to conceive. We found that participants in this study had varied experiences; both positive and negative. However, although some participants reported increased stress when using OTs, they also reported increased stress when trying to conceive without using the tests and many thought that using an OT reduced stress. This finding is in line with the primary finding of the parent study, which compared stress levels in women using an OT with those using no additional aids and found that there was no difference in the level of stress between these groups, as measured by the Perceived Stress Scale, Positive and Negative Affect Schedule questionnaires and biochemical markers of stress [11]. Furthermore, a random sample of 15 participants from each group, from the total of 210 who participated in the parent study, were interviewed at the end of the study. Of these, 67 % of the control group stated that they felt pressure or stress during the study, compared with 13 % of the test group [11].

It was recently reported that many women expect to conceive within a short time frame (≤ 6 months) of starting to try and have little knowledge of the length of their menstrual cycles or when they are ovulating [11]. Many women use the contraceptive pill prior to making the choice to start a family, and thus view cycles as an artificial 28 days, in keeping with the textbook definition of the menstrual cycle. This provides no information concerning the characteristics of the menstrual cycle in individual ovulating women. Women’s menstrual cycles are often variable; the average and median range of cycle variability per woman has been reported as 6–7 days [13, 14]. This lack of understanding is borne out by over half of the estimates from women trying to conceive, who reported their perceived day of ovulation as being outside of their fertile window, and therefore many women trying to conceive may be doing so at the wrong time [6, 8].

There is a rational argument that fertility-focussed intercourse can result in pregnancy being achieved more quickly [5]. The primary results of this intervention study, reported elsewhere, observed more pregnancies among the group of participants randomised to use an OT when trying to conceive compared with the control group who did not use an OT, although this difference did not reach statistical significance (odds ratio: 1.77; 95 % confidence interval [CI]: 0.9992, 3.1585) [11]. Furthermore, the use of a fertility monitor has been found to give an 89 % increase in conception rates across two cycles of use [7]. Therefore, an important finding from our study is that the OT users felt that they had a better understanding of their menstrual cycles and were able to time fertility-focussed intercourse appropriately. It is interesting that we found that the use of OTs also allowed participants to notice changes occurring in their bodies around the time of ovulation. This led to them reporting a better sense of connection with their own fertility.

Obviously, intercourse every 2–3 days would inevitably ensure that intercourse occurs in the fertile window; however, for many couples this is unrealistic. Often couples do not choose to start a family until later in life, when they feel more secure financially, in their employment and in the relationship with their partner. Indeed, the average age for birth of a first child in the UK has gradually increased over the last 3 decades to 29.8 years in 2012 [15]. Increasing levels of sexual dysfunction such as impotence, vaginal dryness and a lack of interest in intercourse have been widely reported with increasing age, and for many couples, intercourse every 2–3 days is unachievable and/or undesirable [16]. Our study findings support this, with participants who followed this guideline also reporting they had no energy or inclination to sustain this and that it also put an added pressure on their relationship, making intercourse less enjoyable and a chore.

A study of 26 patients published by Kopitzke et al. suggested that timing intercourse to coincide with ovulation and the use of OTs could lead to emotional distress [10]. The use of OTs in themselves, however, led to lower levels of distress than most fertility procedures, and significantly lower distress levels than events associated with lack of conception or loss of pregnancy (e.g. negative pregnancy test result, interrupted IVF, onset of menses, ectopic pregnancy and miscarriage). A separate study by Severy et al. investigated acceptability of using a home monitor to measure LH and estrone-3-glucuronide in order to pinpoint fertile days as an aid to conception [3]. This study found that the failure of couples in trying to conceive in successive cycles had a much greater impact on stress than use of the monitor itself.

The study reported here identified that the main advantages of using the digital OT included the ability to pinpoint ovulation and therefore time intercourse, an improved understanding of menstrual cycles, providing support when attempting to conceive, and improving participants’ relationships with their partners. Many participants could not think of any disadvantages of using the OTs, but some mentioned a decline in sexual spontaneity, potential for emotional dependency with continued use, increased pressure on the relationship, and questions and uncertainties which could not be answered easily. One important element of OTs that is often ignored, but was mentioned as a positive benefit by participants in this study, is their negative predictive value – that is, their ability to identify anovular cycles, prompting participants to seek medical advice earlier. This is particularly important when women have delayed starting a family until their 30s, at which time female fertility begins to decline [17], and therefore it is important to obtain assistance in conception as early as possible. Recent studies of cycles from women with normal regular cycles have found an incidence of anovulation of 4.6–7.6 % [18]. Obviously, if an anovular cycle is an isolated occurrence, it is unlikely to have any bearing on a woman’s fertility, but if it recurs across several cycles, it suggests medical intervention would be required to achieve pregnancy.

It is of interest that participants who achieved pregnancy while using OTs appeared to have a lower recollection of how using the test impacted upon their sex life compared with those who did not conceive. This could be a result of non-conceivers having to use the test over a longer period of time, although this information was not collected.

There were some limitations of this study. Participants’ opinions were based on experience of using OTs at the point of interview, which could vary depending on the level of usage prior to study participation, which was not recorded. The results therefore, represent the views of participants who have been using OTs for a variable length of time, the details of which could potentially have been explored in greater depth. Participants were not under clinical investigation for infertility and consequently their experiences may differ to those of women who use OTs while undergoing clinical investigations for infertility. We conducted telephone interviews with the participants and thus only understood the partners’ perspective from the participant’s interpretation of their thoughts and feelings. Although telephone interviews enabled us to interview the geographically diverse study sample with relative ease and cost-effectively, we were not able to observe and respond to any personal signs and expressions of the interviewee, such as facial expression of unease, which can yield important additional information. It has been reported previously that telephone interviews produce data of an inferior quality to face-to-face interviews [19] and that telephone interviews are a less effective method when questions of a personal nature need to be asked and explored [20]. However, given the sensitive nature of this topic, we believe that the respondents may have been more open in their replies due to the anonymity of telephone interviewing. Although some participants reported that they felt stressed when trying to conceive, we did not explore what they explicitly meant by this term. It is recommended that future studies aim to explore the participants’ understanding of the term ‘stress’ and to assess the partner’s experience via direct questioning.

## Conclusions

This study found that the use of home OTs affects women’s thoughts and feelings in multiple ways during attempts to conceive. There were many positive themes: participants not only believed that the test would help them to conceive more quickly and easily, but also that it enhanced their understanding of their own ovarian cycle and reproductive physiology. Ensuring that women who are attempting to conceive have access to appropriate advice is important to help address some of the questions and uncertainties that were raised by the participants in this study. A future study exploring women’s experiences of using OTs when trying to conceive and under clinical investigation for infertility is recommended.

## Additional file

Additional file 1: Table S1. Volunteer individual identification number and demography. (DOCX 24 kb)

## Abbreviations

OT: Ovulation test
LH: Luteinising hormone
NICE: National Institute for Health and Clinical Excellence
RCT: Randomised controlled trial
