# Supplementary material for: Women’s experiences of ovulation testing: a qualitative analysis
Source: Reprod Health. 2015 Dec 21;12:116. doi: 10.1186/s12978-015-0103-y (PMC4687078; doi:10.1186/s12978-015-0103-y)
Supplement: Additional file 1: Table S1. — Volunteer individual identification number and demography. (DOCX 24 kb) [file 12978_2015_103_MOESM1_ESM.docx]

**Additional file 1: Table 1: Volunteer individual identification number and demography.**

| **Participant**  **ID** | **Age** | **Group** | **Number of pregnancies** | **Number of live births** | **Number of miscarriages** | **Months trying**  **to conceive** | **Used ovulation**  **kits before?** | **Pregnancy within**  **study period?** |
| --- | --- | --- | --- | --- | --- | --- | --- | --- |
| 1 | 37 | Control | 1 | 1 | 0 | 12 | Yes | No |
| 2 | 32 | Control | 1 | 0 | 1 | 6 | Yes | Yes |
| 3 | 37 | Test | 2 | 2 | 0 | 3 | Yes | No |
| 4 | 35 | Control | 1 | 1 | 0 | 5 | No | No |
| 5 | 31 | Test | 0 | 0 | 0 | 6 | No | Yes |
| 6 | 35 | Test | 0 | 0 | 0 | 24 | No | No |
| 7 | 36 | Control | 2 | 1 | 1 | 4 | No | No |
| 8 | 28 | Test | 0 | 0 | 0 | 3 | No | Yes |
| 9 | 27 | Test | 1 | 0 | N/G | 6 | No | No |
| 10 | 37 | Test | 1 | 1 | 0 | 6 | No | No |
| 11 | 26 | Test | 1 | 0 | 1 | 2 | No | Yes |
| 12 | 39 | Control | 1 | 1 | 0 | 3 | Yes | No |
| 13 | 31 | Control | 1 | 1 | 0 | 3 | Yes | No |
| 14 | 38 | Test | 1 | 1 | 0 | 4 | Yes | PTP |
| 15 | 25 | Test | 2 | 0 | 2 | 36 | No | No |
| 16 | 26 | Control | 2 | 2 | 0 | 3 | No | No |
| 17 | 31 | Test | 2 | 2 | 0 | 4 | Yes | No |
| 18 | 28 | Test | 0 | 0 | 0 | 2 | Yes | No |
| 19 | 37 | Test | 6 | 1 | 5 | 50 | Yes | No |
| 20 | 27 | Control | 1 | 0 | 1 | 10 | Yes | PTP |
| 21 | 22 | Control | 2 | 1 | 1 | 8 | No | No |
| 22 | 32 | Test | 2 | 2 | 0 | 0 | Yes | No |
| 23 | 28 | Test | 1 | 1 | 0 | 4 | Yes | Yes |
| 24 | 35 | Control | 2 | 1 | 1 | 7 | No | No |
| 25 | 30 | Test | 3 | 2 | 0 | 0 | No | No |
| 26 | 29 | Control | 1 | 1 | 0 | 2 | Yes | Yes |
| 27 | 25 | Control | 2 | 2 | 0 | 8 | Yes | PTP |
| 28 | 32 | Control | 2 | N/G | N/G | 6 | No | Yes |
| 29 | 30 | Control | 2 | 1 | 1 | 4 | Yes | Yes |
| 30 | 33 | Control | 1 | 0 | 1 | 24 | Yes | Yes |
| 31 | 29 | Control | 2 | 0 | 0 | 5 | Yes | Yes |
| 32 | 30 | Test | 1 | 1 | 0 | 5 | Yes | Yes |
| 33 | 35 | Control | 3 | 2 | 1 | 9 | Yes | Yes |
| 34 | 28 | Control | 0 | 0 | 0 | 2 | No | No |
| 35 | 26 | Test | 1 | 0 | 1 | 7 | Yes | Yes |
| 36 | 26 | Test | 0 | 0 | 0 | 11 | No | No |

N/G: not given; PTP: pre-trial pregnancy.
